# Is Childhood IgA Nephropathy Different From Adult IgA Nephropathy? A Narrative Review

**DOI:** 10.1177/20543581251322571

**Published:** 2025-03-12

**Authors:** Areefa Alladin-Karan, Susan M. Samuel, Andrew W. Wade, Pietro Ravani, Silviu Grisaru, Ngan N. Lam, Kathryn A. Bernie, Robert R. Quinn

**Affiliations:** 1 Departments of Pediatrics and Community Health Sciences, Cumming School of Medicine, University of Calgary, AB, Canada; 2 School of Medicine, University of Guyana, Georgetown, Guyana; 3 Department of Pediatrics, The University of British Columbia and BC Children’s Hospital Research Institute, Vancouver, Canada; 4 Department of Pediatrics, Cumming School of Medicine, University of Calgary, AB, Canada; 5 Departments of Medicine and Community Health Sciences, Cumming School of Medicine, University of Calgary, AB, Canada; 6 Department of Anesthesiology, Perioperative and Pain Medicine, and Department of Community Health Sciences, University of Calgary, AB, Canada

**Keywords:** childhood IgA nephropathy, adult IgA nephropathy, disease mechanism

## Abstract

**Purpose of the review:**

Immunoglobulin A (IgA) nephropathy (IgAN) is the most common primary glomerular kidney disease. Children and adults are presumed to have the same disease and are treated similarly. However, there are differences between childhood IgAN and adult IgAN that may require unique treatment considerations, even after transition to adult nephrology services. A narrative review was conducted to compare childhood and adult IgAN and to describe the distinct characteristics of childhood IgAN. Reframing childhood IgAN can inform guideline recommendations unique to childhood IgAN, the development of targeted therapies, and clinical trial design.

**Sources of information:**

Medline and Embase were searched for reports on children and adults with IgAN published between January 2013 and December 2023 (updated May 2024). The search was not restricted by age group, outcomes reported, language, or study design. Randomized controlled trials (RCTs), observational studies, review articles, and nephrology conference abstracts were included. A total of 3104 reports were retrieved. Forty-seven reports (37 primary studies and 10 reviews) were included in the review. Two RCTs and 35 observational studies included a total of 45 085 participants (9223 children and 35 862 adults).

**Method:**

Data were extracted for primary IgAN and not for IgA vasculitis–associated nephritis. Findings were described with no statistical comparisons due to variations in interventions and outcome definitions.

**Key findings:**

Gross hematuria was the obvious clinical difference between childhood IgAN and adult (60-88% vs 15-20%). Nephrotic syndrome was more common in children, approaching up to 44%, while <18% of adults had nephrotic syndrome. Children were biopsied sooner (6 vs 15 months) and had more inflammatory kidney lesions (mesangial hypercellularity: 41-82% vs 38-64%; endocapillary hypercellularity: 39-58% vs 17-34%). Chronic kidney lesions were more prevalent in adults (segmental sclerosis: 62-77% vs 8-51%; interstitial fibrosis/tubular atrophy: 34-37% vs 1-18%). The use of immunosuppressive therapy was higher in children (46-84% vs 35-56%). Children were started on immunosuppressive therapy sooner than adults. Adults were more likely to be optimized with renin-angiotensin system inhibitors (87-94% vs 49-75%). Children had better kidney function than adults at diagnosis (estimated glomerular filtration rate of 90-128 vs 50-88 ml/min/1.73 m^2^), and children also had better kidney survival, with kidney failure of 3.1% vs 13.4% at 5 years. Children had more risk alleles for IgAN and higher levels of mannose-binding lectin than adults.

**Limitations:**

Most studies were retrospective and observational, with limited data on children and disease mechanisms. Data were not pooled for analysis because of important differences in definitions and measurements of baseline characteristics and outcomes. Data from countries with established urine screening programs were different compared to countries without urine screening programs. Some observed differences may be due to practice variation and delayed diagnosis in adults (lead-time bias). Well-designed prospective studies and standardized measures for kidney function assessment and outcomes can reduce heterogeneity and improve results from reviews.

**Conclusion:**

Inherent differences between childhood IgAN and adult IgAN may be due to distinct disease mechanisms. Approaching childhood IgAN as a separate condition could lead to the discovery of targeted therapies and improve management during childhood and after the transition to adult care.

## Abrégé

**Justification:**

La néphropathie à immunoglobulines A (N-IgA) est la néphropathie glomérulaire primitive la plus fréquente. Les enfants et les adultes sont présumés atteints de la même maladie et traités de la même manière. Or, il existe des différences entre la N-IgA de l’enfant et celle de l’adulte susceptibles de nécessiter des considérations particulières en matière de traitement, et ce, même après la transition vers les services de néphrologie pour adultes. Une revue narrative a été réalisée afin de comparer la N-IgA chez l’enfant et l’adulte et de décrire les caractéristiques distinctes de la N-IgA pédiatrique. Une redéfinition de la N-IgA de l’enfant pourrait guider les recommandations des lignes directrices propres à cette population, le développement de thérapies ciblées et la conception d’essais cliniques.

**Sources de l’information:**

Medline et Embase ont été consultées à la recherche d’articles publiés entre janvier 2013 et décembre 2023 (mise à jour en mai 2024) portant sur des enfants et des adultes atteints de N-IgA. La recherche, qui n’a pas été limitée par l’âge, les résultats rapportés, la langue ou la conception de l’étude, a permis de colliger un total de 3 104 articles (essais contrôlés randomisés [ECR], études observationnelles, articles-synthèses et résumés des conférences en néphrologie), desquels quarante-sept (37 études primaires et 10 examens) ont été inclus dans la présente revue narrative. Ces deux ECR et 35 études observationnelles portaient sur un total de 45 085 sujets (9 223 enfants et 35 862 adultes).

**Méthodologie:**

Les données extraites concernaient la N-IgA primaire, et non la néphrite associée à la vascularite à IgA. Les résultats ont été décrits sans comparaison statistique en raison des variations dans les interventions et les définitions des résultats.

**Principaux résultats:**

La présence d’hématurie macroscopique était la principale différence clinique entre la N-IgA de l’enfant et celle de l’adulte (60 à 88 % c. 15 à 20 %). Le syndrome néphrotique était plus fréquent chez les enfants (jusqu’à 44 % des individus) que chez les adultes (moins de 18 %). Les enfants avaient subi une biopsie plus tôt (6 mois c. 15 mois) et présentaient davantage de lésions rénales inflammatoires que les adultes (hypercellularité mésangiale: 41 à 82 % c. 38 à 64 %; hypercellularité endocapillaire: 39 à 58 % c. 17 à 34 %). Les lésions chroniques étaient plus fréquentes chez les adultes (sclérose segmentaire: 62 à 77 % c. 8 à 51 %; fibrose interstitielle/atrophie tubulaire: 34 à 37 % c. 1 à 18 %). L’utilisation d’un traitement immunosuppresseur était plus élevée chez les enfants (46 à 84 % c. 35 à 56 %); ces derniers avaient également amorcé le traitement immunosuppresseur plus tôt. Les adultes étaient quant à eux plus susceptibles d’être traités avec des inhibiteurs du système rénine-angiotensine (87 à 94 % c. 49 à 75 %). Les enfants avaient une meilleure fonction rénale que les adultes au moment du diagnostic (DFGe: 90 à 128 ml/min/1,73 m^2^ c. 50 à 88 ml/min/1,73 m^2^); ils montraient également une meilleure survie rénale, avec 3,1 % d’insuffisance rénale après 5 ans contre 13,4 % pour les adultes. Enfin, les enfants présentaient davantage d’allèles de risque pour la N-IgA et des taux plus élevés de lectine liant le mannose.

**Limites:**

La plupart des études examinées étaient rétrospectives et observationnelles, avec des données limitées chez les enfants et sur les mécanismes de la maladie. Les données n’ont pas été regroupées aux fins d’analyse en raison de différences importantes dans les définitions et les mesures des caractéristiques initiales et des résultats. Les données issues de pays disposant d’un programme établi de dépistage urinaire étaient différentes de celles des pays qui n’en avaient pas. Certaines différences observées pourraient être attribuables à des variations dans les pratiques ou à un diagnostic tardif chez les adultes (biais d’anticipation diagnostique). Des études prospectives bien conçues et des mesures normalisées pour l’évaluation de la fonction rénale et des résultats pourraient réduire l’hétérogénéité et améliorer les résultats tirés des revues narratives.

**Conclusion:**

Les différences intrinsèques entre la N-IgA de l’enfant et celle de l’adulte pourraient s’expliquer par des mécanismes différents de la maladie. Aborder la N-IgA de l’enfant comme étant une maladie distincte pourrait mener à la découverte de thérapies ciblées et améliorer sa prise en charge pendant l’enfance et après la transition vers les soins aux adultes.

## Introduction

Immunoglobulin A (IgA) nephropathy (IgAN) is the most common primary glomerular kidney disease in the world, with an incidence rate ranging from 0.5 to 9.4/100 000 population per year.^1,2^ Immunoglobulin A nephropathy is an immune-mediated disease, and a multi-hit hypothesis has been proposed, wherein there is increased production and circulation of abnormal IgA molecules called galactose-deficient IgA1 (Gd-IgA1; hit 1) that are recognized as antigens and invoke an autoantibody response (hit 2).^2^ These Gd-IgA1 and autoantibodies combine to form immune complexes (hit 3) that deposit in the kidney, causing kidney damage (hit 4).^2^ Dysregulation of the alternative and lectin complement pathways contributes to kidney damage.^3^ The gut mucosa (mucosa-associated lymphoid tissue [MALT]), which has the highest production of IgA molecules, may play a role in hit 1 and hit 2.^4,5^ The hallmark of IgAN is the presence of dominant or co-dominant deposits of IgA molecules in the kidney on immunofluorescence microscopy.^2,6^ The degree of inflammation and scarring (fibrosis) is scored according to the Oxford classification for histopathological staging and prognostication.^6,7^ Immunoglobulin A vasculitis (IgAV), the most common small vessel childhood vasculitis, is a systemic autoimmune disease in which abnormal IgA molecules deposit in multiple organs, including the kidneys.^8^ It causes kidney damage that is indistinguishable from IgAN, known as IgA vasculitis–associated nephritis (IgAVN).^8^ Immunoglobulin A vasculitis–associated nephritis can only be distinguished from IgAN by the presence of systemic signs or symptoms.^8^

Immunoglobulin A nephropathy is a heterogeneous disease.^9^ The prevalence, clinical presentation, and outcomes vary by region (East Asia vs Western), local practices (screening programs, indication for biopsy, and approach to treatment), and age (children vs adults).^5,10,11^ It is most often diagnosed in young adults, but it also affects children and commonly presents with variable degrees of proteinuria and hematuria.^2,5,6^ The outcomes of IgAN are poor, with 30% of adults and 20% of children experiencing a 50% decline in the estimated glomerular filtration rate (eGFR) or developing kidney failure within 10 years.^12-14^

There has been a long-standing assumption that IgAN is the same disease in both children and adults. However, IgAN that presents during childhood (childhood IgAN) may differ from IgAN that presents during adulthood (adult IgAN) in ways that cannot be explained by earlier detection or practice variation.^5,15,16^ It is important to understand these differences as childhood IgAN may have different triggers and disease mechanisms that affect prognosis and require unique treatment considerations.^2,5,16,17^ Applying adult guidelines to children during childhood or when they transition to adulthood may not be ideal as it does not consider the unique characteristics of childhood IgAN. Exploring the differences between childhood and adult IgAN provides an opportunity to reframe childhood IgAN and revisit how children should be treated. A narrative review was conducted to compare childhood and adult IgAN and to describe the distinct characteristics of childhood IgAN. Reframing childhood IgAN can inform (1) guideline recommendations unique to childhood IgAN that include a clinical pathway for continued care after transition to adult nephrology services, (2) the development of targeted therapies, and (3) clinical trial design.

## Methods

Medline and Embase were searched for articles on IgAN that described the prevalence, clinical characteristics, diagnosis, natural history, treatment, and outcomes of children and/or adults. The search was restricted to a 10-year period from January 1, 2013 to December 15, 2023, as the understanding of IgAN has improved significantly during this period. It was updated on May 30, 2024, to include recent publications (Supplemental file 1). The search was not restricted by age group, outcomes reported, language, or study design. Randomized controlled trials (RCTs), observational studies, review articles, and conference abstracts from major nephrology conferences were included (Supplemental file 2, Table S1). Studies that reported on multiple glomerular diseases with specific data IgAN were included. Data were extracted for primary IgAN and not for IgAVN. Data were described with no statistical comparisons because of differences in interventions and outcome definitions.

## Review

**Figure 1.**
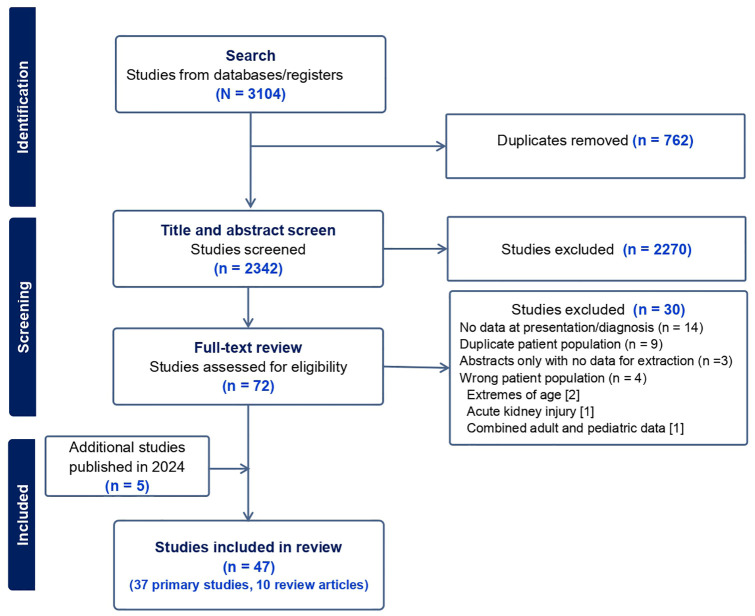
PRISMA diagram of the screening and review process.

A total of 3104 reports were imported into Covidence; 762 duplicates were removed, and 2342 titles and abstracts were screened by one reviewer, of which 2270 were deemed irrelevant, leaving 72 full-text reports that were assessed for eligibility. Five new reports published in 2024 were added after an updated search (Figure 1, Supplemental file 3, Table S2). This narrative review included 47 reports (37 primary studies and 10 reviews on IgAN). Two RCTs and 35 observational studies included a total sample of 45 085 (9223 children and 35 862 adults). Eleven studies were conducted in Japan, 9 in China, 10 in Europe, 3 in the United States, 1 in Saudi Arabia, and 3 were international cohorts. Two non-English articles were included with abstracts and tables published in English, allowing for data extraction. The included articles were summarized based on cohort characteristics at presentation and at biopsy, histopathological findings, treatment, outcomes, and disease mechanisms. Two categories were identified based on the time of kidney biopsy. Childhood IgAN was defined as a diagnosis of IgAN in a child <18 years, except in two studies where an age cut-off of ≤15 years was used, while adult IgAN was defined as a diagnosis of IgAN at ≥18 years of age. Table 1 summarizes the characteristics by age group.

**Table 1.** Baseline Characteristics of Children and Adults Diagnosed With IgA Nephropathy.

Variables	Children (<18 years)	Adults (≥18 years)
Annual incidence^1,10,18,19^	0.2-9/100 000	0.2-4.5/100 000
Primary GN accounted for by IgAN (%)^20,21^	25	34.6
Age at presentation (years, range)^22-26^	10-12.1	38.3
Age at diagnosis (years, range)^13,22,24,25,27-35,36^	10.6-13	33-46
Sex (% males)^13,14,22,25,28,31,32,34,36-38^	50-70	45-76
Family history (%)		
IgAN^34^	18.2	9.6
Any kidney disease^25^	30.5	30.5
Characteristics at time of biopsy		
Time from presentation to biopsy (months)^25^	6	15
eGFR ml/min/1.73 m^2^ (range)^14,22,24-26,28,34,38,39^	89.5-128.4	51.8-87.7
Proteinuria (mg/mmol)^13,34^	210-275	180-241
Hematuria (%)		
Microscopic^25,29,34^	95-100	36-91
Gross^24,37,40,41^	60-88	15-20
Hypertension		
Prevalence (%)^22,24,25,27-29,31^	7-27	22-37
Mean arterial pressure (mmHg)^14,19,35^	85-87 (range)	101 ± 15 (mean, SD)
Systolic blood pressure (mmHg, range)^29,31,34,37,38^	116	124-136
Diastolic blood pressure (mmHg, range)^29,31,34,37,38^	68	74-81
Nephrotic syndrome (%)^28,40,41^	1-35	2-6
Serum albumin (g/l)^22,25,26,34,40^	33-38	38-39
Oxford score		
M1 (range in %)^14,24,26,31,36,37,40,42,43^	41-82	38-64
E1 (range in %)^14,24,26,31,36,37,40,42,43^	39-58	17-34
S1 (range in %)^14,24,26,31,36,37,40,42,43^	8-51	62-77
T1/T2 (range in %)^14,24,26,31,36,37,40,42,43^	1-18	34-37
C1/C2 (range in %)^14,24,26,31,36,37,40,42,43^	28-52	34-59

*Note.* eGFR = estimated glomerular filtration rate; GN = glomerulonephritis; IgAN = IgA nephropathy; M = mesangial hypercellularity; E = endocapillary hypercellularity; S = segmental sclerosis; T = tubular atrophy/interstitial fibrosis; C = crescents.

### Section 1: Differences Between Children and Adults With IgAN

#### Incidence

The incidence and distribution of IgAN varied based on several factors, including age, screening, and diagnostic practices. In regions where school urine screening was established, such as Japan, the annual incidence of IgAN was 4.5 to 9/100 000 in children under 15 years of age and 3.9 to 4.5/100 000 in adults.^1,10^ In other parts of the world, where routine urine screening was not done, the annual incidence ranged from 0.2 to 0.6/100 000 in children and 0.2 to 1.9/100 000 in adults.^10,19,18^ The incidence of IgAN peaked in adolescents and young adults.^44^ Immunoglobulin A nephropathy represented 50% of primary glomerulonephritis diagnoses in adolescents and young adults (15-30 years) and 25% in children <15 years in Japan, with lower distributions seen in other regions (34.6% in adults from Canada and 9.3% in children from Saudi Arabia).^19-21^ Findings from regions with school urine screening programs suggested that there were childhood-onset and adult-onset forms of IgAN, as individuals in Japan who were diagnosed with adult IgAN would presumably have had negative urine screens during childhood.

#### Symptoms and signs

##### Hematuria

Hematuria is the most common clinical feature in IgAN and is a marker of disease activity.^45,46^ Hematuria was the most common presenting feature of IgAN in children and could be either microscopic or gross.^15,47^ While microscopic hematuria was almost universal in children and adults, gross hematuria was seen in only 15% to 20% of adults compared with 70% to 88% of children.^37,39,48,49^ Persistent and higher levels of microscopic hematuria were associated with poor kidney outcomes.^24,45,46,50^ Hematuria in children is probably the most important finding that triggers an investigation for a glomerular disease and a diagnosis of IgAN.^48^ In Japan, 70% to 80% of pediatric cases were detected by abnormal urine screens that showed microscopic hematuria/proteinuria and 15% to 20% presented with gross hematuria.^11,51^ Most children will have one or more episodes of gross hematuria, resulting in a medical encounter and investigations for a glomerular disease.^24^ This typical presentation in children allows for an earlier diagnosis of IgAN.^24^ Glomerular hematuria without persistent severe proteinuria (0.5-1 g/day) does not typically trigger a biopsy in adults, delaying diagnosis of IgAN.^29,46^

Compared with persistent microscopic hematuria, recurrent episodes of gross hematuria were associated with less proteinuria and improved kidney survival in children and adults, although the underlying reason for this is not established.^45,46,50-52^ Microscopic hematuria was associated with inflammatory kidney lesions, and achieving remission of microscopic hematuria (<5 RBC/hpf) indicated a favorable kidney prognosis.^24,45^ Gross hematuria in children may indicate distinct disease pathogenesis compared to adults and has implications for diagnosis and prognosis. Gross hematuria is usually concurrent with respiratory or gastrointestinal infections and may be related to Gd-IgA1 and antibody levels.^4,24,46,53^

##### Proteinuria and nephrotic syndrome

Proteinuria is an indicator of disease severity and an independent predictor of kidney outcomes at diagnosis and during follow-up.^54,55^ Proteinuria was higher in children than adults and reached statistical significance in some studies that compared IgAN between children and adults (1.8 vs 1.3 g/day, *P* < .001).^13,15,49^

Nephrotic syndrome at diagnosis is indicated by significant proteinuria (≥200 mg/mmol or ≥1000 mg/m^2^/day) and low serum albumin (<30 g/l).^56^ Serum albumin levels were lower in children.^22,28,49,57^ Higher proteinuria and lower serum albumin levels in children corresponded to a higher frequency of nephrotic syndrome at the time of biopsy, although this was not statistically different in most studies and ranged from 1% to 10%.^28,48,58,59^ However, in one study from China, nephrotic syndrome frequency approached 35% in children, compared with 6% in adults (*P* < .001).^49^ The etiology of proteinuria in children and adults seemed to differ. Inflammation, which was more common in children, probably led to glomerular basement membrane damage and subsequent proteinuria, which was associated with mesangial and endocapillary hypercellularity.^15,60^ On the other hand, adults had more chronicity at the time of diagnosis, and proteinuria was similar to that seen in progressive chronic kidney disease (CKD), being associated with glomerulosclerosis, tubular atrophy, and lower eGFR.^15,60^ Immunoglobulin A nephropathy can co-exist with minimal change disease, with biopsy findings showing effacement of podocyte foot processes and IgA deposits, which has been described as IgAN-minimal change disease.^61,62^ It is unclear if these are two co-existing diseases or minimal change disease with IgA deposits.^61,63^ Minimal change disease is more common in children, which may explain the higher frequency of nephrotic syndrome in children with IgAN.

Proteinuria, regardless of the cause, is an important predictor of prognosis and is toxic to kidney tubules, leading to further inflammation and fibrosis.^54,60^ Therefore, a reduction of proteinuria is beneficial for both children and adults, but this may require different management strategies based on histology. Children have more proteinuria, higher eGFR, and inflammatory kidney lesions, which favor early immunosuppressive therapy in attempts to preserve kidney function and reduce scarring, while proteinuria in adults due to chronic kidney lesions (fibrosis and tubular atrophy) is not amenable to immunosuppressive therapies and is best treated with optimal supportive care to reduce nephron loss.^6,9^

##### Hypertension

Hypertension at diagnosis is associated with poor kidney outcomes.^14,36,27^ The prevalence of hypertension at the time of biopsy increased with age and ranged from 7% to 12% in children to 63% to 71% in older adults.^25,28,37,58,31,64^ Adults <65 years had a prevalence of 22% to 37%, except for TESTING cohort, where the prevalence was 48%.^25,28,37,58,65^ Most pediatric studies reported mean arterial pressures, systolic blood pressure (SBP), and diastolic blood pressure (DBP), which cannot be interpreted clinically, as these numbers change with age, height, and sex.^66^ The frequency of hypertension in children is probably a more practical measure when summarizing studies. Blood pressures were not compared with non-IgAN patients or a healthy control group in any of the reviewed studies. Hypertension in children was mostly due to kidney disease and was associated with older age, lower eGFR, and more sclerosis compared with non-hypertensive children with IgAN.^27^

The high prevalence of hypertension in older adults was most likely due to co-morbid conditions such as essential hypertension or cardiovascular disease, although it could have been caused by IgAN.^65,67^ While the frequency of hypertension was higher in older adults, blood pressures at diagnosis were similar between younger and older adults, probably due to intensive blood pressure control in older adults who were more likely to be on multiple medications for hypertension.^65,68^

The duration, severity, and type of hypertension affect kidney outcomes, which are not specific to IgAN.^67,69^ Arterial stiffness, which increases with age, is an important cause of systolic hypertension in CKD patients, typically manifesting with increased SBP, reduced DBP, and elevated pulse pressure, as was documented in older patients with IgAN.^65,67,70^ Hypertension can damage the kidney microvasculature, leading to a faster decline in kidney function and worse outcomes, particularly in older patients.^27,65,67^

##### Estimated glomerular filtration rate

Children had higher eGFR at presentation and at biopsy compared with adults in all cohorts evaluated. CureGN reported a median eGFR of 98.6 ml/min/1.73 m^2^ in 173 children and 51.8 ml/min/1.73 m^2^ in 333 adults at the time of biopsy (*P* < .001).^47^ The range of eGFR across all studies at biopsy was 89 to 117 ml/min/1.73 m^2^ in children, 52 to 88 ml/min/1.73 m^2^ in younger adults, and 29 to 47 ml/min/1.73 m^2^ in older adults.^14,15,37,47,65,68,71-73^ Estimated glomerular filtration rate at diagnosis predicts treatment and prognosis.^6,14,36^ Higher eGFR in children indicates an opportunity to preserve kidney function and reduce inflammation, which may warrant immunosuppressive therapy early in the disease. Adults, on the other hand, have lower eGFR, often indicating more chronic changes, which are likely to be less responsive to immunosuppressive therapies, thus favoring the optimization of supportive care.^6^

#### Diagnosis

##### Time to biopsy

Diagnosis of IgAN relies on a kidney biopsy.^6^ Time from presentation to biopsy differed between children and adults. Presentation referred to clinical detection, symptoms or signs consistent with a glomerular disease, or a presumed clinical diagnosis of IgAN.^24,47^ An important finding, based on limited data, was that children were biopsied earlier in their clinical course than adults (6 vs 15 months after presentation).^28,47,48,57,71^ Several factors influenced the time to biopsy, including practice variation and access to nephrology and biopsy services.^29^ Children tend to have a clearer presentation of IgAN with recurrent gross hematuria that triggers visits to the emergency department and early consultation with pediatric nephrology services.^49^ Therefore, pediatric nephrologists see children earlier in their disease course. Additionally, pediatric nephrologists may have a lower threshold for performing kidney biopsies and starting immunosuppressive therapy in children who present with glomerulonephritis, especially with persistent proteinuria.^16,47^ Immunoglobulin A nephropathy in adults is more likely to have a silent presentation detected on routine medical exams with a urine screen by a family physician.^11,29^ Investigation of hematuria in adults is more extensive and sometimes involves urology services, which further delays nephrology encounters.^50^ Adult nephrology services have higher patient volumes and longer wait times that may delay encounters, resulting in a longer time to biopsy.^29,74^ Reduced access to adult nephrology services was associated with a longer time to biopsy and more severe disease at diagnosis.^29^ Severe clinical disease was inversely associated with the number of nephrologists per 100 000 regional population in a large Japanese study of registry data.^29^ In addition, adult nephrologists may optimize supportive therapy in presumed IgAN before proceeding to biopsy or considering immunosuppressive therapy, which may also explain the delay in performing a kidney biopsy.^47^ The survival time for children may seem longer if they are diagnosed early in the course of the disease because of more clinical features that declare themselves, like gross hematuria. This phenomenon is known as lead-time bias.^75^

##### Histopathological differences

Pathological findings in reviewed studies were reported according to the Oxford classification.^7,76^ The mean percentages of each lesion (mesangial hypercellularity [M], endocapillary hypercellularity [E], segmental sclerosis [S], tubular atrophy/interstitial fibrosis [T], and crescents [C]) were reported using MEST-C scores. Children had more inflammatory lesions (M and E) than adults who had more chronic (S and T) lesions, which corresponded to lower eGFR. Crescents were most common in children (28-52%).^14,36,48,57,68,71^ Inflammatory lesions (M and E) in children corresponded to higher eGFR and proteinuria and were more likely to improve with immunosuppressive therapy.^57^ Children presenting with gross hematuria had more inflammatory lesions and better kidney outcomes.^45,46,52^

#### Treatment

Treatment recommendations for children and adults with IgAN are similar and follow the Kidney Disease Improving Global Outcomes (KDIGO) 2021 Glomerulonephritis Guideline.^6^ Optimization of supportive therapy, including maximum tolerated renin-angiotensin system inhibitor (RASi) and control of hypertension, is recommended for all patients.^6,77^ Immunosuppressive therapy is recommended for those with active disease on histology and proteinuria who do not have adequate response to supportive care.^6,77^

##### Supportive care

Children were less likely to be on RASi at the time of a kidney biopsy than adults (11% vs 34%, respectively, Table 2).^14,36^ Children were also less likely to be placed on RASi at any point during their disease course compared with adults (49-67% and 88-94%, respectively).^25,40,42,71^ However, there was no difference in the timing of initiation of RASi after biopsy between adults and children when they were used (1.4 and 1.6 months, respectively).^14,36^ Optimization of supportive care, including RASi, was lower in children than adults, although the evidence is strong for optimizing supportive care for everyone diagnosed with IgAN, regardless of age.^12,40,78^ This difference was probably due to a shorter time to kidney biopsy with less time for optimizing RASi, a greater reliance on immunosuppressive therapy, and a lack of clinical practice guidelines for children with IgAN.^16,17,78^

**Table 2.** Treatment and Outcomes of Children and Adults Diagnosed With IgA Nephropathy.

Variables	Children (<18 years)	Adults (≥ 18 years)
Treatment		
RASi at biopsy (%)^14,36^	11.1	32.4
RASi during follow-up (%)^14,36^	63.4	86.7
RASi at any time (%)^24,25^	49-75	88.6-94
Immunosuppression before biopsy (%)^14,36,40^	14.1	9.1
Immunosuppression after biopsy (%)^14,36^	58.1	43.5
Any immunosuppression (% in range)^25,26,35,39,42^	46-84	35-56
Time to immunosuppression start post-biopsy (months)^14,36^	0.8	1.6
Steroids (% of patients treated)^24,25,40^	46.2-87	45-53.2
Mycophenolate (% of patients treated)^25^	14.5	10.8
Azathioprine (% of patients treated)^25^	5.2	3.9
Cyclophosphamide (% of patients treated)^25^	4.5	4
Outcomes		
Clinical remission (%)^22,79^	52	12.2
eGFR decline, annual (ml/min/1.73 m^2^)^35,13^	0 to −0.8 (mild) −3.6 (severe)	−2.4 to −4.97
Kidney failure (%)^14,13,36^	3.1 (5-year follow-up) 44 (13.4-year follow-up)	13.4 (5-year follow-up) 67 (7.7-year follow-up)
Kidney survival^13^	5 years: 91 10 years: 76 20 years: 52	5 years: 71 10 years: 55 20 years: 28
Time to kidney failure (years)^13^	21.6	10.8
Age at kidney failure (years)^13^	27	49

*Note.* eGFR = estimated glomerular filtration rate; RASi = renin-angiotensin system inhibitor.

##### Immunosuppression

The majority of patients with IgAN, regardless of age, received immunosuppressive therapy, and corticosteroids (steroids) were the most commonly prescribed agent (Table 2).^14,33,36,40,42,47^ Children were more likely to be treated with immunosuppressive therapy than adults (46-84% and 35-56%, respectively).^14,36^ Although the use of immunosuppressive therapy before a kidney biopsy was infrequent, children were more likely to receive immunosuppressive therapy before a confirmed biopsy diagnosis of IgAN compared with adults.^14,36^ Children were also more likely to receive immunosuppressive therapy much sooner after a kidney biopsy than adults (0.8 vs 1.6 months, respectively).^14,36^

Clinical and histological characteristics might explain differences in the use of immunosuppressive therapy in children and adults. Children had more inflammatory kidney lesions, including crescents, which were amendable to treatment with steroids, with improved proteinuria and eGFR.^6,57,78^ Although adults had more chronicity and lower eGFR, a significant proportion received immunosuppressive therapy in an attempt to stop any inflammation and preserve kidney function.^15,80^ This practice is common in both adult and pediatric nephrology practices, as IgAN has poor kidney outcomes.^78^

The shorter time to initiation of immunosuppression in children was probably due to the nature of pediatric nephrology services, as biopsy reports are typically available sooner, and pediatric nephrologists are more inclined to start steroids.^78,81^ Pediatric nephrologists are more likely to start steroids around the time of RASi treatment initiation to induce remission of proteinuria and stop inflammation.^78^ Adult nephrologists may optimize supportive care for 3 to 6 months before starting steroids as per the STOPIgAN and TESTING protocols.^37,72^ Unlike in adults, there are no RCTs in children outside of Japan to guide the timing of initiation, dose, and duration of steroid therapy.^16,17,82^ Additionally, the indications for steroid therapy in children are not well established.^16,78^ Steroid use before biopsy in children could be explained by the fact that children presenting with nephrotic syndrome are routinely treated empirically with steroids for presumed minimal change disease without biopsy confirmation.^56,78,83^

A minority of patients received multiple immunosuppressive agents. Mycophenolate mofetil was the second most commonly prescribed immunosuppressive agent (12.1%), followed by azathioprine (4.3%) and cyclophosphamide (4.3%).^25^ Mycophenolate use was higher in children than adults (14.5% vs 10.8%).^47^ The higher use of steroid-sparing immunosuppressive medications in children was probably to avoid steroid toxicity when long-term immunosuppressive therapy was desired. Steroids impair growth, reduce bone mineral density, and cause weight gain, which can significantly affect pubertal children during accelerated growth and development.^84^ Children may sometimes be treated with immunosuppressive therapy for up to 2 years and even longer, and it is important to consider the toxicity profile of immunosuppressants.^16^ In the MAIN RCT, mycophenolate mofetil combined with supportive care, or supportive care only, was continued for at least 18 months in adults from China.^85^ The composite outcome of doubling of serum creatinine, kidney failure, or death due to kidney or cardiovascular disease occurred in 7.1% in the mycophenolate group and 21.2% in the supportive care group (adjusted hazard ratio 0.23; 95% confidence interval, 0.09-0.63), with no increased toxicity. Mycophenolate may be an alternative immunosuppressive medication when steroid toxicity is a concern. The recently published (September 2024) International Pediatric Nephrology Association (IPNA) Guideline for the management of IgAN and IgAVN in children has detailed recommendations for supportive and immunosuppressive therapy, and KDIGO is updating the glomerulonephritis guideline (2024 Draft) to incorporate findings from recent studies.^77^ Briefly, the IPNA guideline suggested steroids in those at risk of progression, such as (1) persistent proteinuria ≥50 mg/mmol despite RASi use, (2) active MEST-C scores, and (3) crescentic IgAN. Other immunosuppressive agents, including mycophenolate and cyclophosphamide, were suggested for rapidly progressive IgAN, nephrotic syndrome, and for those who did not respond to steroids.

#### Outcomes

The impact of age on kidney outcomes in patients with IgAN is not well established as many factors may affect outcomes, such as disease severity at diagnosis, treatment regimens, and co-morbid conditions.^13,86^ Measures of kidney outcomes varied among studies. Some reported on eGFR decline and kidney failure, while others reported on clinical remission and proteinuria reduction.

##### eGFR decline and kidney failure

Immunoglobulin A nephropathy was associated with progressive decline in eGFR and kidney failure, which were reported as annual change in eGFR, development of kidney failure, and kidney survival (Table 2). Annual eGFR loss in children was lower and ranged from 0 to 0.8 ml/min/1.73 m^2^ per year for milder kidney disease at diagnosis and 3.6 ml/min/1.73 m^2^ per year for severe disease at diagnosis.^13,35,69^ Annual eGFR loss was higher in younger adults and nearly 3 times higher in older adults.^13,37,64^ Adults progressed to kidney failure, dialysis, transplant, or death 50% sooner than children (13.6 vs 6.6 years, respectively).^13^ The prognosis was worst in older adults, with half developing kidney failure or dying within 5 years of diagnosis.^68^ Kidney failure prevalence was highest among children and adults with a history of significant proteinuria (>0.5 g/day) or reduced eGFR (<60 ml/min/1.73 m^2^) at diagnosis.^13^ A recent study reported that kidney failure occurred in 44% of children followed for 13.4 years, and 67% of adults followed for 7.7 years.^13^ Children had a median kidney survival of 21.6 years with age at kidney failure of 27 years, and adults had a median kidney survival of 10.8 years with age at kidney failure of 49 years.^13^ Of note, this study excluded mild cases of IgAN in both children and adults.^13^

##### Proteinuria reduction and clinical remission

Comparison of proteinuria reduction and clinical remission across studies was challenging due to heterogeneous definitions and treatment protocols/interventions. Some studies defined remission as a 25% or greater reduction in proteinuria, an absolute reduction to <1 g/day, or no proteinuria (urine proteinuria to creatinine ratio <20 mg/mmol), while others included remission of hematuria (<5 RBC/hpf) and/or stable eGFR (≥90 ml/min/1.73 m^2^) in a composite endpoint referred to as clinical remission. Children and adults who achieved proteinuria reduction to <1 g/day or experienced remission appeared to have better kidney outcomes.^22,55^ Kidney disease progression was predicted by the degree of proteinuria at baseline and during follow-up, regardless of age.^22,55^ Clinical remission was higher in children (Table 2). Two Japanese studies reported 52% remission in 100 children, followed for 11.8 years and 12.2% in 74 adults, followed for 7.8 years.^22,79^ An Italian study on 153 children reported clinical remission at 10 years of 43%.^24^ Although this study only included children, older age at disease onset and chronic lesions were associated with reduced clinical remission, while immunosuppressive therapy did not seem to influence the likelihood of clinical remission.^24^

##### Recurrence after transplant

Immunoglobulin A nephropathy will recur in up to 60% of patients after a kidney transplant.^87^ Younger age at diagnosis and particularly childhood IgAN had a higher risk of recurrence.^87-90^ Graft loss due to recurrent disease was significantly higher in children with parental living donors versus non-parental living donors.^91^ A faster decline to kidney failure and more crescents in native biopsies were also associated with recurrence.^89^ This suggests that the immune mechanisms that trigger early-onset IgAN may also be implicated in recurrent IgAN, and some of these mechanisms are outside of the kidney and are inherited.

### Section 2: Reasons Why IgAN Is Different Between Children and Adults

Children present differently from adults. Data from countries with urine screening programs support the existence of childhood IgAN and adult IgAN with distinct characteristics. Childhood IgAN is more likely to present with gross hematuria, nephrotic syndrome, higher eGFR, and inflammatory kidney lesions. Adult-onset IgAN has milder proteinuria, persistent microscopic hematuria (subclinical), and more chronic kidney lesions. These differences may reflect different disease mechanisms between children and adults, practice variations, and lead-time bias associated with timing of diagnosis.

#### Disease mechanisms

##### Genetics

Immunoglobulin A nephropathy is a heterogeneous disease with a genetic predisposition, dysregulation of the immune system, and environmental triggers. Risk alleles involved in the integrity of the gut mucosa and the glomerular basement membrane have been identified.^5,92,93^ Children, especially those with a family history of IgAN, had more risk alleles compared to adults.^5^ Variants in type IV collagen (COL4A3 and COL4A4) were identified in children with early-onset IgAN. COL4A3 variants in children were associated with severe IgAN, recurrent flares, gross hematuria, and faster decline in kidney function.^92^ These variants may explain recurrent gross hematuria in children with concurrent infections (synpharyngitic hematuria), similar to Alport syndrome.^92^ Since gross hematuria is the key clinical feature distinguishing childhood IgAN from adult IgAN, variants in type IV collagen may be the distinct disease mechanism explaining this difference.

##### Galactose-deficient IgA1

Children with IgAN had lower levels of Gd-IgA than adults with IgAN, but they had significantly higher levels of Gd-IgA1 compared with healthy pediatric controls.^39,94^ Galactose-deficient IgA1 levels did not predict clinical presentation or pathology findings.^94,95^ Since children have lower levels of Gd-IgA1, other factors probably increase Gd-IgA1 deposition in the kidney. One postulated mechanism is that children with IgAN and COL4A variants may have thinning of the glomerular basement membrane, increasing the permeability of the kidney to Gd-IgA1.^96^ Another mechanism that may be more common in children is the trigger of IgAN with infections of the respiratory and gastrointestinal tract.^5,97^ Infections of the mucosa can increase the production of IgA in the MALT, leading to spikes in Gd-IgA1 levels in susceptible children and overt presentation or flares with gross hematuria.^5^ Susceptibility in these children is probably linked to COL4A variants or risk alleles involved in gut mucosa integrity or failure of the immune system to regulate Gd-IgA1 levels.^5,92,93^

##### Complement pathway

The alternative and lectin pathways are activated in IgAN as indicated by positive staining in the kidney for C3, C4d, and Manose-binding lectin (MBL) and negative staining for C1q (absence of activation of the classical pathway).^2,3^ Children had significantly higher levels of circulating MBL, higher glomerular deposition of terminal complement (C5b-9), and higher intensity of C4d staining, compared with adults.^39,42,93^ Higher intensity of C4d positive stains was associated with higher levels of proteinuria and more mesangial hypercellularity and sclerosis in children.^41^

##### Infections

Children are more susceptible to infections than adults, and mucosal infections can trigger overt clinical presentation or flares of IgAN, often with gross hematuria.^5,98^ Infections can increase the susceptibility of children to IgAN through multiple mechanisms. First, *Streptococcus pyogenes* commonly infects the respiratory mucosa of children and expresses M protein, which can bind to Gd-IgA1 and was identified in the kidneys of children with IgAN.^98^ The M proteins increased proliferation in the mesangium and C3 production beyond levels observed with Gd-IgA1 alone.^98^ This may explain why chronic tonsillitis increased the risk of developing IgAN in a large cohort from Japan.^99^ Second, mucosal infections may be the main trigger for increased production of Gd-IgA1 in the MALT.^4,5,98^ Third, children with IgAN have more risk alleles that affect the integrity of the intestinal mucosa than adults, which may lead to increased exposure to antigens and increased Gd-IgA1 production in susceptible children.^5,93^

**Figure 2.**
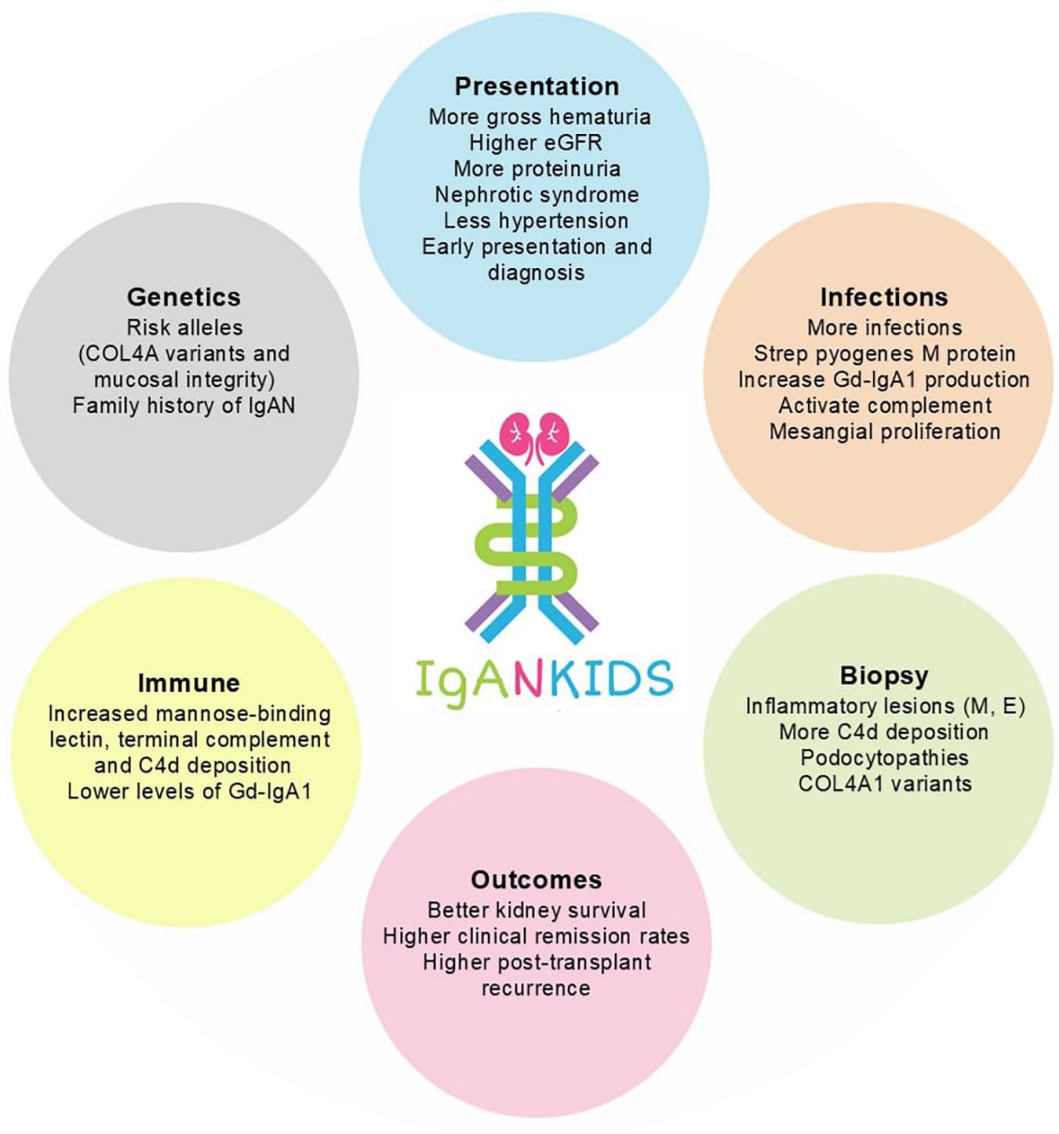
Typical features of childhood IgAN (compared with adult IgAN). *Note.* COL4A1 = collagen type IVA1; eGFR = estimated glomerular filtration rate; Gd-IgA1 = galactose-deficient immunoglobulin A1; GN = glomerulonephritis; IgAN = IgA nephropathy.

In summary, childhood IgAN may have distinct disease mechanisms that lead to kidney damage. Children have more risk alleles and more activation of the alternative and lectin pathways than adults.^39,42,93^ Children are also more susceptible to infections, which can increase Gd-IgA1 levels (Figure 2). It is important to consider these distinctions between childhood and adult IgAN when inferring data from adult studies to treat children with IgAN. These differences are important considerations when choosing therapy. Further work to determine if childhood IgAN is distinct from adult IgAN is warranted to better understand mechanisms of IgAN and develop targeted therapies.

#### Practice variation

Variations in practice by geographic region and between adult and pediatric nephrology services impact some of the observed differences in IgAN between children and adults.^10,82^ Established urine screening programs improve early detection and diagnosis, which can improve outcomes.^11^ Children presenting with hematuria/proteinuria are more likely to be investigated for a primary glomerular kidney disease as compared with adults who have a broader list of differential diagnoses.^50^ This may delay nephrology encounters and diagnosis for adults. Access to nephrology and biopsy services also differ and adult nephrology services have longer wait times.^29^ The approach to treatment of IgAN differs between pediatric and adult nephrology services.^16,78^ A higher prevalence of inflammatory kidney lesions in children coupled with the drive to reduce disease progression may favor earlier biopsies and the use of immunosuppressive therapies.^78^ There is a lack of evidence to guide treatment in children, while there is strong evidence to guide the optimization of supportive therapy before considering immunosuppressive therapy in adults.^16,17,77,78,82^ Support is a major factor influencing outcomes for patients with CKD.^16,74,81^ Care for children is typically caregiver-led, and caregivers advocate for children, which may improve adherence and outcomes, compared with adults.^16,74,81,100^

#### Lead-time bias

Children had higher eGFR, less chronic changes in the kidney, and better kidney survival than adults. The absence of gross hematuria in adults and less obvious symptoms, along with factors affecting biopsy timing, such as practice differences between pediatric and adult nephrologists may lead to a later diagnosis when there is more kidney damage and a shorter kidney survival time. The delay in diagnosis may also mean that adults are less responsive to immunosuppressive therapies because of the presence of more chronic damage. However, lead-time bias cannot fully explain the differences between childhood and adult IgAN and some of these differences may be explained by distinct disease mechanisms.

#### Transition to adult nephrology services

If childhood IgAN has a different disease mechanism from adult IgAN, treatment after transitioning should follow a unique pathway with targeted therapies for childhood IgAN, and children who transition should not necessarily be treated as per adult IgAN guidelines. There is no clear direction from KDIGO or IPNA for managing childhood IgAN during adulthood. Adults with IgAN diagnosed during childhood and adults with a history of hematuria and proteinuria during childhood require consideration in clinical trial design and targeted therapy development, as their treatment responses may reflect the mechanisms of childhood IgAN and can inform recommendations for children in the absence of pediatric trials.

## Limitations

Most of the included studies were retrospective and observational and were characterized by important heterogeneity in definitions, kidney function assessment, and outcome measures. Treatment varied widely between children, adults, and by regions, making it challenging to merge the data and make comparisons. Data from countries with established urine screening programs were different compared to countries without urine screening programs. Data on children with IgAN were sparse, limiting the interpretation of the findings of this review and any statistical comparisons between children and adults. Patients with presumed mild IgAN without a biopsy-confirmed diagnosis were excluded from these studies. Therefore, the findings from this review may not apply to that population. Some cohorts may be duplicated because it was challenging to identify all reports with different titles that analyzed the same cohorts. Well-designed prospective studies and standardized measures for kidney function assessment and outcomes could reduce heterogeneity and improve comparability of studies going forward.^82^

## Conclusion

Inherent differences between childhood IgAN and adult IgAN may be due to distinct disease mechanisms. Differences in complement pathway activation, COL4A variants, and podocytopathies could explain certain clinical characteristics such as gross hematuria and nephrotic syndrome in children. Approaching childhood IgAN as a separate condition could lead to the discovery of targeted therapies and improve management during childhood and after the transition to adult care. Children with IgAN and adults with a history of childhood IgAN require special consideration, especially when developing practice recommendations and designing clinical trials.

## Supplemental Material

sj-docx-1-cjk-10.1177_20543581251322571 – Supplemental material for Is Childhood IgA Nephropathy Different From Adult IgA Nephropathy? A Narrative Review Supplemental material, sj-docx-1-cjk-10.1177_20543581251322571 for Is Childhood IgA Nephropathy Different From Adult IgA Nephropathy? A Narrative Review by Areefa Alladin-Karan, Susan M. Samuel, Andrew W. Wade, Pietro Ravani, Silviu Grisaru, Ngan N. Lam, Kathryn A. Bernie and Robert R. Quinn in Canadian Journal of Kidney Health and Disease

sj-docx-2-cjk-10.1177_20543581251322571 – Supplemental material for Is Childhood IgA Nephropathy Different From Adult IgA Nephropathy? A Narrative Review Supplemental material, sj-docx-2-cjk-10.1177_20543581251322571 for Is Childhood IgA Nephropathy Different From Adult IgA Nephropathy? A Narrative Review by Areefa Alladin-Karan, Susan M. Samuel, Andrew W. Wade, Pietro Ravani, Silviu Grisaru, Ngan N. Lam, Kathryn A. Bernie and Robert R. Quinn in Canadian Journal of Kidney Health and Disease

sj-docx-3-cjk-10.1177_20543581251322571 – Supplemental material for Is Childhood IgA Nephropathy Different From Adult IgA Nephropathy?: A Narrative Review Supplemental material, sj-docx-3-cjk-10.1177_20543581251322571 for Is Childhood IgA Nephropathy Different From Adult IgA Nephropathy?: A Narrative Review by Areefa Alladin-Karan, Susan M. Samuel, Andrew W. Wade, Pietro Ravani, Silviu Grisaru, Ngan N. Lam, Kathryn A. Bernie and Robert R. Quinn in Canadian Journal of Kidney Health and Disease
