# Corrigendum to “Is childhood IgA nephropathy different from adult IgA nephropathy? A narrative review”

**DOI:** 10.1177/20543581251359782

**Published:** 2025-07-22

Alladin-Karan A, Samuel SM, Wade AW, et al. Is Childhood IgA Nephropathy Different From Adult IgA Nephropathy? A Narrative Review. *Canadian Journal of Kidney Health and Disease*. 2025;12. doi:10.1177/20543581251322571

One of the authors of this article Kathryn A. Birnie reported that their name was misspelt in the article. This correction has no influence on the original experiments and maintains the scholarly validity and integrity of the article.

The article has been updated with the correct name of that author.

