# Supplementary material for: Is Childhood IgA Nephropathy Different From Adult IgA Nephropathy? A Narrative Review
Source: Can J Kidney Health Dis. 2025 Mar 12;12:20543581251322571. doi: 10.1177/20543581251322571 (PMC11898040; doi:10.1177/20543581251322571)
Supplement: sj-docx-1-cjk-10.1177_20543581251322571 – Supplemental material for Is Childhood IgA Nephropathy Different From Adult IgA Nephropathy? A Narrative Review [file sj-docx-1-cjk-10.1177_20543581251322571.docx]

**Supplemental file 1: Search strategy outcomes for the narrative review of children and adults diagnosed with IgA Nephropathy**

Search Date: 15^th^ Dec 2023

Databases: Medline and Embase

Search strategy

1. Glomerulonephritis, IgA/
2. Iga glomeruloneph$.tw,kf.
3. Berger$ disease.tw,kf.
4. Iga nephropathy$.tw,kf.
5. IgAN.tw,kf.
6. IgAGN.tw,kf
7. igA-N.tw,kf
8. immunoglobulin a nephropathy.tw,kf.
9. Or/1-8
10. Differenc$.tw,kf.
11. Gap$.tw,kf.
12. Compar$.tw,kf.
13. Or/10-12
14. Exp child/
15. Exp adolescent/
16. Exp youth/
17. Exp pediatrics
18. Exp school
19. Or/14-18
20. Adult$.tw,kf.
21. Elder$.tw,kf
22. Or/20-21
23. and/9,13,22

This search yielded 5794 abstracts. When the publication year was limited to 2013-current, 3104 abstracts remained, which were imported into Covidence
