# Supplementary material for: Is Childhood IgA Nephropathy Different From Adult IgA Nephropathy? A Narrative Review
Source: Can J Kidney Health Dis. 2025 Mar 12;12:20543581251322571. doi: 10.1177/20543581251322571 (PMC11898040; doi:10.1177/20543581251322571)
Supplement: sj-docx-2-cjk-10.1177_20543581251322571 – Supplemental material for Is Childhood IgA Nephropathy Different From Adult IgA Nephropathy? A Narrative Review [file sj-docx-2-cjk-10.1177_20543581251322571.docx]

Supplemental file 2

**Table S1: Inclusion and exclusion criteria for the narrative review of children and adults diagnosed with IgA Nephropathy**

| **Criteria** | **Characteristics** |
| --- | --- |
| Inclusion criteria | Population: Patients with primary IgA nephropathy  Studies: Trials, observational studies, systematic reviews on IgA nephropathy  Variables: Studies that reported on characteristics at presentation or at biopsy and the inclusion and exclusion criteria for individual studies  Date: studies published from 2013 to May 2024 |
| Exclusion criteria | Population: Secondary IgA nephropathy, IgA vasculitis with nephritis, all other glomerular diseases and systemic diseases, and hospitalized patients  Age categories: Studies that defined adults as age < 45-50 years old and studies that reported only combined pediatric and adult data were excluded  Studies: Case studies and case series, review articles that did not present data, commentaries, and protocols  Outcomes: acute kidney injury, outcomes that are not validated or do not have strong evidence to support use, e.g. urine proteomics biomarkers |
