# Supplementary material for: Is Childhood IgA Nephropathy Different From Adult IgA Nephropathy? A Narrative Review
Source: Can J Kidney Health Dis. 2025 Mar 12;12:20543581251322571. doi: 10.1177/20543581251322571 (PMC11898040; doi:10.1177/20543581251322571)
Supplement: sj-docx-3-cjk-10.1177_20543581251322571 – Supplemental material for Is Childhood IgA Nephropathy Different From Adult IgA Nephropathy?: A Narrative Review [file sj-docx-3-cjk-10.1177_20543581251322571.docx]

Supplemental file 3

**Table S2: Included studies in the narrative review of children and adults diagnosed with IgA Nephropathy**

| **Studies** | | **Title** | **Comments** |
| --- | --- | --- | --- |
| Primary studies | | | |
| **Japan (n=11)** | Kamei 2016 | Proteinuria during Follow-Up Period and Long-Term Renal Survival of Childhood IgA Nephropathy | 100 children |
|  | Koike 2024 | Clinicopathological prognostic stratification for proteinuria and kidney survival in IgA nephropathy: a Japanese prospective cohort study | 991 adults |
|  | Komatsu 2016 | Clinical manifestations of Henoch–Sch ̈ onlein purpura nephritis and IgA nephropathy: comparative analysis of data from the Japan Renal Biopsy Registry (J-RBR) | 803 children, 4876 adults |
|  | Matsushita 2015 | Long-term morbidity of IgA nephropathy in children evaluated with newly proposed remission criteria in Japan | 53 children ≤ 15 years |
|  | Matsuzaki 2021 | Utility of remission criteria for the renal prognosis of IgA nephropathy | 74 adults |
|  | Moriyama 2014 | Prognosis in IgA Nephropathy: 30-Year Analysis of 1,012 Patients at a Single Center in Japan | 1012 adults |
|  | Okabayashi 2018 | Distribution of nephrologists and regional variation in the clinical severity of IgA nephropathy at biopsy diagnosis in Japan: a cross-sectional study | 6426 adults |
|  | Okabayashi 2016 | Reduction of proteinuria by therapeutic intervention improves the renal outcome of elderly patients with IgA nephropathy | 87 adults ≥ 60 years |
|  | Tanaka 2013 | Development and Validation of a Prediction Rule Using the Oxford Classification in IgA Nephropathy | 698 adults |
|  | Tsunoda 2019 | Characteristics of IgA nephropathy in the elderly: Results from a multicenter, large-scale, long-term observational cohort study | 151 adults ≥ 60 years |
|  | Urushihara 2021 | Clinical and histological features in pediatric and adolescent/young adult patients with renal disease: a cross-sectional analysis of the Japan Renal Biopsy Registry (J-RBR) | 3463 children < 15 years, 6532 adolescents and young adults (15-30 years) |
| **China (n=9)** | Fu 2020 | Hypertension/prehypertension and its determinants in pediatric IgA nephropathy | 108 children |
|  | Jiang 2021 | Clinical and immune characteristic differences between children and adult-onset IgA nephropathy (abstract) | 70 children, 280 adults |
|  | Jiang 2015 | Serum galactose-deficient IgA1 levels in children with IgA nephropathy | 72 children |
|  | Lv 2022 | Effect of Oral Methylprednisolone on Decline in Kidney Function or Kidney Failure in Patients With IgA Nephropathy: The TESTING Randomized Clinical Trial | 503 adults |
|  | Siyu Dai 2023 | Comparison of clinical and pathological characteristics between children and adults with primary IgA nephropathy and its clinical significance | 160 children, 240 adults |
|  | Su 2024 | Are Children with IgA Nephropathy Different from Adult Patients? | 1015 children,1911 adults |
|  | Wen 2020 | Clinical, pathological characteristics and outcomes of immunoglobulin A nephropathy patients with different ages | 981 adolescents and adults (≥ 14 years) |
|  | Xu-hui 2023 | Differences between children and adults with primary IgA nephropathy and associated mechanism (IPNA abstract) | 996 children,1768 adults |
|  | Zhou 2023 | Association between glomerular C4d deposition, proteinuria, and disease severity in children with IgA nephropathy | 65 adults |
| Europe (n=10) | Antonucci 2024 | Childhood-onset IgA Nephropathy: Is Long-term Recovery Possible? | 153 children |
|  | Cambier 2021 | Rare Collagenous Heterozygote Variants in Children With IgA Nephropathy | 36 children |
|  | Cambier 2020 | Clinical and histological differences between adults and children in new-onset IgA nephropathy | 82 children, 129 adults |
|  | Coppo 2017 | Risk Factors for Progression in Children and Young Adults with IgA Nephropathy: An Analysis of 261 Cases from the VALIGA European Cohort | 87 children, 174 adults (< 23 years) |
|  | Mizerska-Wasiak 2016 | IgA Nephropathy in Children: A Multicenter Study in Poland | 140 children |
|  | Pitcher 2023 | Long-Term Outcomes in IgA Nephropathy | 140 children, 2299 adults |
|  | Rauen 2015 | Intensive Supportive Care plus Immunosuppression in IgA Nephropathy-Protocol | 337 adults |
|  | Rodas 2020 | IgA Nephropathy Recurrence after Kidney Transplantation: Role of Recipient Age and Human Leukocyte Antigen-B Mismatch | 86 adults |
|  | Sevillano 2019 | IgA Nephropathy in Elderly Patients ≥65 years | 151 adults |
|  | Sevillano 2016 | IgA Nephropathy (IgAN) in Patients over 64 Years Old: A Devastating Disease with No Effective Treatment | 142 adults |
| **US A** | Avasare 2017 | Predicting Post-Transplant Recurrence of IgA Nephropathy: The Importance of Crescents | 62 adults |
|  | Cheungpasitporn 2015 | Primary IgA nephropathy in elderly patients | 207 adults |
|  | Engen 2024 | Risk for graft loss in pediatric and young adult kidney transplant recipients due to recurrent IgA nephropathy | 343 children, 1572 adults < 25 years |
| International cohort | Barbour 2021 | Updating the International IgAN Prediction Tool for use in children | 1060 children |
|  | Barbour 2019 | Updating the International IgAN Prediction Tool for use in children | 3927 adults |
|  | Selewski 2018 | Clinical Characteristics and Treatment Patterns of Children and Adults with IgA Nephropathy or IgA Vasculitis: Findings from the CureGN Study | 173 children, 333 adults |
| Arab | Alhasan 2020 | Renal histopathology spectrum in children with kidney diseases in Saudi Arabia, 1998-2017 | 17 children with IgAN (total cohort = 326) |
| **Review articles** | | | |
| Canney 2020 | | Socioeconomic Position and Incidence of Glomerular Diseases | |
| Coppo 2021 | | What progress has been made in understanding IgA Nephropathy in children in the last decade? | |
| Coppo 2020 | | IgA nephropathy in children and in adults: two separate entities or the same disease? | |
| Doherty 2022 | | Systematic Review on Health-Related Quality of Life and Utilities in Immunoglobulin A Nephropathy | |
| Fabiano 2016 | | Immunoglobulin A nephropathy: Pathological markers of renal survival in paediatric patients | |
| Honda 2024 | | Cost-Effectiveness of School Urinary Screening for Early Detection of IgA Nephropathy in Japan | |
| Kiryluk 2023 | | Global Incidence of IgA Nephropathy by Race and Ethnicity: A Systematic Review | |
| Kiryluk 2014 | | The genetics and immunobiology of IgA nephropathy | |
| Schena 2018 | | Epidemiology of IgA Nephropathy: A Global Perspective | |
| Willey 2023 | | The incidence and prevalence of IgA nephropathy in Europe | |

Footnote: Studies were assigned a country if > 70% of the sample were from that country
